# Liver cell circuits and therapeutic discovery for advanced liver disease and cancer

**DOI:** 10.5802/crbiol.64

**Published:** 2021-11-15

**Authors:** Emilie Crouchet, Catherine Schuster, Thomas F. Baumert

**Affiliations:** a Université de Strasbourg, Inserm, Institut de Recherche sur les Maladies Virales et Hépatiques UMR_S1110, F-67000 Strasbourg, France; b Institut Hospitalo-Universitaire, Pôle Hépato-digestif, Nouvel Hôpital Civil, F-67000 Strasbourg, France; c Institut Universitaire de France, Paris, France

**Keywords:** Hepatocellular carcinoma, Chemoprevention, Epigenetic, Gene signature, Risk prediction, Fibrosis, Carcinome hépatocellulaire, Chimioprévention, Épigénétique, Signature génétique, Prédiction du risque, Fibrose

## Abstract

Hepatocellular carcinoma (HCC) is a major global health challenge with rising incidence. Despite the previous approval of several novel therapeutic approaches, HCC remains the second common cause of cancer-related death worldwide. The vast majority of HCCs arises in the context of chronic fibrotic liver diseases caused by viral or metabolic etiologies. In patients with advanced liver disease the risk of HCC persists even after viral cure or control of infection. Moreover, given the change in the lifestyle and increase of obesity and metabolic disorders, HCC incidence is predicted to drastically augment in the next decade. Early detection, improvement of the screening method in patient at-risk and development of chemopreventive strategies are therefore urgently needed to reduce HCC risk. This review summarizes the major challenges in the identification of patient at risk for HCC and the emergent strategies for HCC prevention to improve patients’ outcome.

## Résumé

Le carcinome hépatocellulaire (CHC) est un problème de santé mondial majeur dont l’incidence est en constante augmentation. Malgré l’approbation de plusieurs nouvelles approches thérapeutiques, le CHC demeure la deuxième cause de décès par cancer dans le monde. La grande majorité des CHC survient dans le contexte de maladies chroniques fibrotiques du foie causées par des étiologies virales ou métaboliques. Chez les patients présentant une maladie hépatique avancée, le risque de CHC persiste même après l’élimination du virus ou le contrôle de l’infection virale. En outre, compte tenu du changement de mode de vie et de l’augmentation de l’obésité et des troubles métaboliques, l’incidence du CHC devrait augmenter de façon spectaculaire au cours de la prochaine décennie. La détection précoce, l’amélioration des méthodes de dépistage chez les patients à risque et le développement de stratégies chimiopréventives sont donc nécessaires de toute urgence pour réduire le risque de CHC. Cette revue résume les principaux défis dans l’identification des patients à risque pour le CHC et les stratégies émergentes pour la prévention du CHC afin d’améliorer la prise en charge des patients.

## 1 Introduction

Advanced liver diseases and hepatocellular carcinoma (HCC), the major type of liver cancer, are a major challenge for global health affecting more than 20% of the EU population. HCC is the second leading and fastest rising cause of cancer-related death worldwide and the leading cause of death among cirrhotic patients [1]. The European Association for the Study of the Liver (EASL) reported that HCC is responsible for 70,000 deaths/year in Western countries. It is estimated that 85–95% of the HCCs arise in the context of chronic fibrotic liver diseases, mainly due to chronic hepatitis B and C, alcoholic liver disease and non-alcoholic steatohepatitis (NASH), the most severe form of non-alcoholic fatty liver disease (NAFLD) [1, 2]. In Europe, 70% of the cases can be attributed to chronic hepatitis C. Despite the development and widespread use of direct-acting antivirals (DAAs) which have revolutionary improved the management of hepatitis C virus (HCV) infection, the risk of HCC development in patient with advanced fibrosis persists even after a decade of viral cure [3]. Moreover, given the change in the lifestyle and the increasing of metabolic disorders, obesity and diabetes, NAFLD and NASH are predicted to be a major cause for HCC in the future [2]. HCC incidence will therefore keep increasing in the coming decades.

Current treatment options for HCC are still unsatisfactory. Only 30–40% of HCC patients are eligible to curative surgical approaches (resection, ablation, transplantation) because they are often diagnosed in advanced stage with poor liver function. Moreover, about 70% of the patients experience tumor recurrence within 5 years after surgical resection of ablation [1]. While several new compounds for HCC treatment have been recently approved, the overall response rate remains limited. The most recent combination of Vascular endothelial growth factor (VEGF)-targeting agents with immune checkpoint inhibitors targeting programmed cell death 1 (PD-1) is characterized by response rates remaining at less than 30% [4]. Despite emerging molecular targeted therapies for HCC, identification of novel targets has been daunting tasks due to the complexity and heterogeneity of cancer initiation and development mechanisms [5]. Prevention of HCC development and progression in patients at risk has therefore emerged as a promising strategy to decrease the overall HCC disease burden. In this review, we summarize the challenge to identify patients at risk for HCC, review current HCC chemoprevention approaches and discuss the challenges and limitations in HCC prevention.

## 2 HCC chemoprevention: roadblocks and challenges

### 2.1 HCC screening and prediction

Given the limited therapeutic options currently available for HCC, prevention and early detection in at-risk individuals remain the most important strategy. Despite many advances, some challenges and roadblocks remain. Clinical practice guidelines recommend biannual HCC screening in at-risk patients for early tumor detection using ultrasound with or without *α*-fetoprotein (AFAP) detection [6]. Meta-analysis studies showed that early tumor detection is significantly associated with a higher probability of benefiting from a curative treatment and so a higher survival probability in patients. Early detection can also help to decrease the economic burden of HCC [7, 8]. However, it appears that HCC screening is underutilized, with a rate lower than 20%, because of patient and provider-related barriers [6]. Given the rising numbers of patients with advanced liver fibrosis, the vast number of patients to be screened and the emergence of novel populations at-risk (i.e. NASH patients and HCV-cured patients) constitute another major barrier [9]. Therefore, prediction of HCC risk in patient may help to improve HCC screening by distinguishing high-risk populations eligible for HCC chemoprevention from low-risk populations for long term vigilance. Nowadays, reliable tools to predict HCC risk, tumor recurrence and treatment response are still absent. Only the Barcelona Clinic Liver Cancer (BCLC) prognostic system was validated by EASL clinical guidelines, which classifies patients and recommend treatment accordingly [10]. However, this system has some limitations due to intermediate profiles making the choice of the best treatment often challenging.

To address this unmet medical need, several biomarker candidates have been developed based on genome wide expression profiling of patient liver tissues to predict the clinical outcome and HCC risk in patient with advanced liver disease. The most widely studied biomarker is a pan-etiology 186-gene clinical prognostic liver signature (PLS) in diseased liver tissues robustly predicting liver disease progression, patient outcome, HCC risk and tumor recurrence in multiple patient cohorts [11-15]. A reduced version of the PLS, comprising 32 bioinformatically selected and clinically validated genes, was recently implemented in an FDA-approved diagnostic platform for clinical use as Laboratory Developed Test (LDT) [16]. Moreover, a blood-based non-invasive signature comprising 8 soluble proteins, the PLSsec, has been developed as a surrogate of the PLS to accurately predict HCC risk in patient with advanced fibrosis [17]. Another signature based on genome wide epigenetic changes in the liver induced by viral and metabolic liver disease has been recently suggested to predict HCC risk [18, 19]. Jühling *et al*. uncovered a 25 gene prognostic epigenetic signature termed PES robustly predicting HCC risk and survival in patients with metabolic and viral liver disease [19]. The PES reflects the presence of epigenetic dysregulation appearing in patients with chronic liver disease which drive hepatocarcinogenesis by altering gene expression [18, 19].

In association with clinical scoring systems and biomarkers (i.e. AFAP), the PLS, the PLSec and the PES provide a perspective to improve patient care, HCC risk stratification and surveillance. Moreover, they may also help clinical testing of chemopreventive compounds by enrolling high-risk patients and at the same time decreasing clinical trial costs.

### 2.2 Target and drug discovery

Another major challenge is the identification of clinically relevant targets. Development of chemopreventive strategies has been daunting tasks due to (i) the complexity of cancer initiation and development mechanisms (ii) the difficulty to validate targets in patients (iii) the absence of reliable and simple models reflecting the cell circuits relevant for HCC development [1, 9].

A “reverse-engineering” approach has been recently developed in order to identify relevant targets in clinical cohorts with completed long-term followup, before validation in experimental models [15]. Advancing this concept to a platform for drug and target discovery, Crouchet *et al*. developed a simple and robust human cell culture system that models the clinical PLS (described above) in an inducible and reversible manner (cPLS system). This system recapitulates the cell circuits relevant for liver disease progression and HCC development in a simplified manner for all the major etiologies [19–21]. In contrast to other models, this system is based on a clinically relevant readout and multitarget approach (186 genes). It offers the opportunity to validate clinical targets and to discover anti-fibrotic compounds for HCC chemoprevention by reversing the PLS from a poor- to a -good-prognosis. This model was recently used as a novel and simple drug discovery platform by screening computationally prioritized candidate compounds currently approved for long-term clinical use without severe adverse effects. The cPLS model, followed by validation in animal models, allowed the discovery of nizatidine, a histamine receptor H2 (HRH2) blocker, for treatment of fibrotic liver disease and HCC chemoprevention [21]. Mechanistic studies demonstrated that nizatidine prevents HCC by decreasing liver inflammation, preventing fibrosis development and through direct anti-cancer properties. Discovery of nizatidine as a novel anti-fibrotic compound for HCC prevention demonstrates the validity and translatability of the cPLS model [21].

As described above, chronic liver disease induces epigenetic reprogramming in patients associated with hepatocarcinogenesis. Epigenetic changes, mainly consisting in hypermethylation or acetylation of histones, results in perturbation of gene transcription. It was demonstrated that epigenetic changes are correlated with induction of the poor-prognosis PLS associated with HCC risk in patients [19]. The bromodomain and motif extraterminal (BET) proteins are chromatin readers which bind histone acetylation and promote oncogene upregulation by recruiting transcription complexes [22] (Figure 1). BET proteins are overexpressed in several solid tumors and play a key role in hepatocarcinogenesis [22]. Interestingly, the BET inhibitor JQ1 has been shown to reverse both the poor-prognosis PLS and PES in the cPLS model [19]. Moreover, JQ1 had a marked impact on fibrosis in rodent model, restored transcriptional program in the liver and prevented HCC development [19]. BET inhibitors were therefore suggested as a novel strategy for HCC chemoprevention [23] (Figure 1).

Validation of anti-fibrotic compounds for chemoprevention also requires robust and easy to use patient-derived models to validate therapeutic approaches predicting clinical success. To address this challenge, Thomas Baumert’s laboratory developed a patient-derived spheroid system harboring the main liver cell compartments and reflecting the liver micro-environment, in which, the clinical PLS can be modeled and reversed by candidate compounds [21]. In the future, we can imagine implementing cost-effective clinical testing of candidate compounds by combining target and drug discovery pipeline with reverse-engineering approaches and HCC biomarkers to risk-stratify the patients.

## 3 HCC chemoprevention strategies

Cancer prevention strategies are based on different interventions: primary, secondary, and tertiary preventions (Figure 2). Primary prevention mainly consists in limiting the risk factors. It is estimated that 40 to 45% of cancers are attributable to preventable risk factors including tobacco, alcohol, physical inactivity, and unbalanced diet. While the incidence of cancer is increasing worldwide, prevention appears as a key strategy to reduce this burden [24, 25]. Primary prevention of HCC mainly focuses on lifestyle modification and HBV vaccination. Secondary prevention is the identification and treatment of premalignant or malignant processes through screening, early detection, and effective treatment in patient already exposed to etiological agents. Tertiary prevention aims to reduce cancer recurrence after treatment or *de novo* carcinogenesis in the pro-carcinogenic milieu [5, 24] (Figure 2).

### 3.1 HCC prevention in metabolic liver disease

About 25% of the global population suffers from NAFLD. NAFLD is strongly associated with metabolic syndrome including obesity, dyslipidemia and type 2 diabetes mellitus [2]. Obesity and high body-mass index (BMI) are directly associated with HCC risk by driving steatosis, chronic inflammation, insulin resistance and oxidative stress [2]. AASLD guidelines recommend HCC surveillance in NAFLD patient every 6 months. However, abdominal ultrasound in obese patients is challenging [6]. Mounting evidence have shown that targeting metabolic abnormalities by either nutritional or pharmaceutical intervention may be an effective strategy to prevent HCC in obese and NAFLD patients. The primary preventive strategy for NAFLD patient consists in lifestyle changes through diet and exercise, as they benefit the full pathogenic spectrum of NAFLD and reduce progression of liver damage. Pharmaceutical intervention is mainly focused on modulation of metabolic pathways and inflammation using metformin and statins [2, 26]. Metformin is an inhibitor of the AMP-activated protein kinase used in patient with diabetes. It has been associated with a reduction of HCC risk most likely by reducing oxidative stress and hyperinsulinemia [27, 28]. Statins are broadly used as cholesterol lowering compounds. However, several studies have suggested an anti-HCC effect independent of this effect. Statins were shown to inhibit hepatocarcinogenesis by targeting different HCC drivers (i.e. Myc, NF-*κ*B, Akt) by decreasing inflammation and by reducing fibrogenesis [5]. Statins may therefore have a HCC protective effect in at-risk patient regardless of the etiology but can also be used as tertiary intervention to prevent recurrence.

### 3.2 Chronic hepatitis B and persistence of HBV cccDNA

Chronic HBV infection accounts for the development of more than 50% of the HCCs in the world. HBV vaccination program has successfully reduced the number of chronic HBV carriers and is effective as a primary HCC prevention. However, chronic HBV infection is still a major cause of liver disease in unvaccinated patients, in particular in Southeast Asia and sub-Saharan Africa [29]. According to the World Health Organization (WHO), more than 240 million people are chronically infected with HBV. These individuals are at risk for HCC and represent a target population for secondary prevention. Antiviral therapies targeting HBV replication have been evaluated for HCC prevention. Retrospectives studies reported HCC risk reduction after suppression of HBV replication with the new generation of nucleot(s)ide analogs (i.e. entecavir and tenofovir) [30, 31]. However, despite significant reduction, the HCC risk cannot be eliminated. The surveillance in patient with chronic HBV infection is therefore mandatory. HBV DNA integration into the host genome as well as the viral covalently closed circular DNA (cccDNA) are unique features of the HBV life cycle likely contributing to carcinogenesis. Next generation therapeutic approaches aiming to completely eradicate HBV including elimination of the viral cccDNA may help to improve prevention of HCC development in the future [32].

### 3.3 Chronic hepatitis C and persistent HCC risk post DAA cure

Chronic HCV infection leads to fibrosis, cirrhosis and associated complication including liver failure and HCC. It corresponds to the major HCC etiology in Western countries and Japan [1]. Globally, 71 million persons are infected. The “baby boomers” represent the specific population with the highest rates of chronic HCV infection and related-HCC mortality because most of the individuals were infected before the discovery of HCV and the development of the modern detection techniques [1]. Despite the development of the DAAs which have enabled an improvement of HCV clearance, the HCV-related HCC incidence is predicted to dramatically increase in the next decades. DAAs use is still hampered by limited access and chronic hepatitis C is often undiagnosed because chronic disease progresses during decades without symptoms. Moreover, studies in large clinical cohorts have shown that despite decrease in HCC risk after sustained virologic response, patients with advanced liver fibrosis remain at high risk for HCC [3]. Recent studies published by Hamdane *et al*. have shown that chronic HCV infection induces epigenetic alterations associated HCC risk which persist even after DAA cure [18]. Epigenetic alterations are therefore attractive targets to prevent HCC in at-risk patients treated for HCV infection. Moreover, in a proof-of-concept study, Jühling *et al*. showed that inhibition of the chromatin reader BRD4 using a small molecule JQ1 prevents liver fibrosis progression toward HCC by restoring transcriptional reprogramming of the genes epigenetically altered in patients [19] (Figure 1). HCV-induced HCC is often diagnosed at a late stage due to the long and asymptomatic progression of chronic liver disease. As the development of a prophylactic HCV vaccine is still challenging [33], identifying the patients with HCC high risk is likely to improve management of the patients and prevention of HCC.

### 3.4 HCC chemopreventive approaches: examples of clinical studies

Chronic inflammation and liver fibrosis are well established drivers of HCC and therefore constitute attractive targets for HCC chemoprevention [1]. However, there is an enormous unmet medical need for liver fibrosis and antifibrotic therapies are not yet available. Major efforts in drug and target discovery are underway to treat fibrotic liver disease and prevent HCC development. Several generic molecules targeting inflammation and fibrosis entered in clinical trials and are also evaluated for HCC prevention (for a review, see [9]). Recently, the use of non-steroidal anti-inflammatory drugs such as aspirin in patients was shown to be associated with a decrease of hepatocarcinogenesis and HCC risk [34]. This anticancer effect may be due to the anti-inflammatory properties of aspirin though inhibition of the NF-*κ*B signaling. Different studies also suggested a direct inhibition of P4HA2, a key enzyme of the collagen synthesis. However, other studies have shown an antiplatelet effect of aspirin limiting its clinical application in patients with chronic liver disease [34, 35]. More studies are needed to decipher the exact mechanism of action.

Multiple signaling pathways have been identified as drivers of hepatocarcinogenic and cell proliferation and became attractive for generic HCC prevention. As example, the inhibitors or the phosphoinositide 3-kinase (PI3K)/AKT/mTOR pathway, sirolimus or everolimus are now investigated for HCC recurrence in phase III clinical trials [36]. Erlotinib, the inhibitor of the epidermal growth factor receptor (EGFR) is also entering in phase I clinical trial following preclinical studies which demonstrated that erlotinib prevents HCC development *in vivo* and reverses the poor-prognosis PLS associated with HCC risk [9, 37]. All these strategies may be of utmost interest to prevent preneoplastic lesions but also to prevent HCC recurrence after resection or transplantation by targeting the pro-carcinogenic milieu, which persists in the injured liver, even after viral cure.

## 4 Conclusions and perspectives

HCC is a major cause of cancer-related death worldwide which is predicted to increase in the next decades. Despite tremendous progress, curative treatments are still unsatisfactory. The vast majority of HCC patients are diagnosed at an advanced stage because the populations at risk are not clearly defined. Improvement of patient surveillance and screening as well as development of HCC chemopreventive strategies will therefore ameliorate patient management and prognosis in the future. Promotion of lifestyle change by healthcare professionals may significantly decrease HCC incidence. Moreover, combining predictive gene signature and/or HCC biomarkers will help to risk stratify the patient and establish effective and accurate therapeutic strategy for HCC prevention. The development of target discovery pipeline and patient-derived models coupled with reverse-engineering approaches will help to develop novel chemopreventive strategies. Discovery of safe generic compounds reducing liver inflammation and fibrosis may contribute to the development of cost-effective HCC chemoprevention strategies. As HCC can arise on early stage of even non-fibrotic liver in particular in the context of chronic HBV infection or NASH, these approaches may be associated with etiology-specific intervention to efficiently prevent HCC development. Chemoprevention is expected to substantially improve management of patient at risk for HCC.

## French version

### 1 Introduction

Les maladies hépatiques avancées et le carcinome hépatocellulaire (CHC), le principal type de cancer du foie, constituent un défi majeur pour la santé mondiale, touchant plus de 20 % de la population de l’Union européenne. Le CHC est la deuxième cause de décès liés au cancer dans le monde en constante et rapide augmentation, et la première cause de décès chez les patients cirrhotiques [1]. Selon *l’European Association for the Study of the Liver* (EASL, association européenne pour l’étude du foie), le CHC est responsable de 70 000 décès par an dans les pays occidentaux. Il est estimé que 85 à 95 % des CHC surviennent dans le contexte de maladies chroniques fibrotiques du foie, principalement dues aux hépatites chroniques B et C, aux maladies alcooliques du foie et à la stéatohépatite non alcoolique (*non alcoholic steatohepatitis*, NASH), la forme la plus grave de la stéatose hépatique non alcoolique (*non alcoholic fatty liver disease*, NAFLD) [1, 2]. En Europe, 70 % des cas peuvent être attribués à l’hépatite C chronique. Malgré le développement et l’utilisation généralisée des antiviraux à action directe (AAD) qui ont révolutionné la prise en charge de l’infection par le virus de l’hépatite C (VHC), le risque de développer un CHC chez les patients présentant une fibrose avancée persiste plus d’une décennie après l’élimination du virus [3]. En outre, compte tenu du changement de mode de vie et de l’augmentation des troubles métaboliques, del’obésité et du diabète, la NAFLD et la NASH représenteront une cause majeure de CHC à l’avenir [2]. L’incidence du CHC continuera donc à augmenter dans les décennies à venir.

Les options thérapeutiques actuelles pour le CHC sont encore insatisfaisantes. Seuls 30 à 40 % des patients atteints de CHC peuvent bénéficier d’une approche chirurgicale curative (résection, ablation, transplantation) car le CHC est souvent diagnostiqué à un stade avancé de la maladie, lorsque la fonction hépatique est déjà fortement détériorée. En outre, environ 70 % des patients connaissent une récidive tumorale dans les 5 ans suivant la résection chirurgicale ou l’ablation [1]. Bien que plusieurs nouveaux composés pour le traitement du CHC aient été récemment approuvés, le taux de réponse global reste limité. La récente combinaison d’agents ciblant le *Vascular endothelial growth factor* (VEGF) et des inhibiteurs de points de contrôle immunitaires ciblant la protéine *Programmed cell death 1* (PD-1) a montré des taux de réponse inférieurs à 30 % [4]. Malgré l’émergence de thérapies moléculaires ciblées pour le CHC, l’identification de nouvelles cibles est une tâche ardue en raison de la complexité et de l’hétérogénéité des mécanismes d’initiation et de développement du cancer [5]. La prévention du développement et de la progression du CHC chez les patients à risque est donc apparue comme une stratégie prometteuse pour réduire la progression de ce fardeau. Dans cette revue, nous résumons le défi que représente l’identification des patients à risque pour le CHC, nous passons en revue les approches actuelles de chimioprévention du CHC et nous discutons des défis et des limites de la prévention du CHC.

### 2 Chimioprévention du CHC : obstacles et défis

#### 2.1 Dépistage et prédiction du CHC

Compte tenu des options thérapeutiques limitées actuellement disponibles pour le CHC, la prévention et la détection précoce chez les personnes à risque restent la stratégie la plus intéressante. Malgré de nombreuses avancées, certains défis et obstacles subsistent. Les directives de pratique clinique recommandent un dépistage semestriel du CHC chez les patients à risque pour une détection précoce de la tumeur par échographie avec ou sans détection del’*α*-fœtoprotéine (AFAP) [6]. Des études de méta-analyse ont montré que la détection précoce de la tumeur est significativement associée à une probabilité plus élevée de bénéficier d’un traitement curatif et donc à une probabilité de survie plus élevée chez les patients. La détection précoce peut également contribuer à diminuer la charge économique liée au CHC [7, 8]. Cependant, il semble que le dépistage du CHC soit sous-utilisé, avec un taux inférieur à 20 %, tant à cause des patients qu’aux praticiens [6]. Étant donné le nombre croissant de patients atteints de fibrose hépatique avancée, le nombre conséquent de patients à dépister et l’émergence de nouvelles populations à risque (c’est-à-dire les patients atteints de NASH et les patients guéris d’une hépatite C chronique) constituent un autre obstacle majeur [9].

Par conséquent, la prédiction du risque de CHC chez le patient pourrait aider à améliorer le dépistage du CHC en distinguant les populations à haut risque éligibles pour la chimioprévention du CHC des populations à faible risque pour une vigilance à long terme. Aujourd’hui, il n’existe toujours pas d’outils fiables pour prédire le risque de CHC, la récidive tumorale et la réponse au traitement. Seul le système pronostique de la clinique du cancer du foie de Barcelone (BCLC), permettant de stratifier les patients et recommandant un traitement approprié, a été validé par les directives cliniques de l’EASL, [10]. Cependant, ce système présente certaines limites en raison de l’existence de profils intermédiaires, rendant le choix du meilleur traitement souvent difficile.

Pour répondre à ce besoin médical, plusieurs bio-marqueurs ont été identifiés grâce à l’étude transcriptomique de tissus hépatiques de patients, afin de prédire l’évolution de la maladie, la réponse à un traitement et le risque de CHC chez les patients atteints d’une maladie hépatique avancée. Le biomarqueur le plus largement étudié est une signature hépatique pronostique (*prognostic liver signature*, PLS) de 186 gènes identifiée dans les tissus hépatiques des patients quelle que soit l’étiologie de la maladie. La PLS prédit de manière robuste la progression de la maladie hépatique, la survie du patient, le risque de CHC et la récidive tumorale dans de multiples cohortes de patients [11–15]. Une version réduite de la PLS, comprenant 32 gènes sélectionnés grâce à des études bio-informatiques et validés cliniquement, a récemment été implémentée dans une plateforme de diagnostic approuvée par la *Food and Drug Administration* (FDA), pour une utilisation en clinique (*Laboratory Developed Test* ou LDT) [16]. De plus, l’identification d’une signature dite « soluble » et non invasive comprenant 8 protéines détectables dans le sang, la PLS-sec, a été développée comme un « clone » de la PLS pour prédire avec précision le risque de CHC chez les patients présentant une fibrose avancée [17]. Une autre signature basée sur les changements épigénétiques induits par les maladies hépatiques virales et métaboliques à l’échelle du génome dans le foie a récemment été suggérée pour prédire le risque de CHC [18, 19]. Jühling *etal*. ont découvert une signature épigénétique pronostique de 25 gènes appelée PES (*prognostic epigenetic signature*) prédisant de manière robuste le risque de CHC et la survie chez les patients atteints de maladies hépatiques métaboliques et virales [19]. La signature épigénétique pronostique reflète la présence de dérèglements épigénétiques apparaissant chez les patients souffrant de maladies chroniques du foie, qui favorisent l’hépatocarcinogenèse en modifiant l’expression des gènes [18, 19].

En association avec les systèmes de classification clinique et les biomarqueurs (par exemple l’AFAP), la PLS, la PLSec et la PES offrent des perspectives intéressantes pour améliorer les soins aux patients, la stratification du risque de CHC et la surveillance de la récidive. En outre, ils peuvent également contribuer à l’élaboration d’essais cliniques pour des composés chimiopréventifs, en permettant de recruter de patients à haut risque (population ciblée) et en réduisant de ce fait leurs coûts.

#### 2.2 Découverte de cibles thérapeutiques et de médicaments

Un autre défi majeur est l’identification de cibles thérapeutiques cliniquement pertinentes. Le développement de stratégies de chimioprévention est une tâche ardue en raison (i) de la complexité des mécanismes d’initiation et de développement du cancer, (ii) de la difficulté à valider les cibles chez les patients et (iii) de l’absence de modèles fiables et simples reflétant les circuits cellulaires menant au développement du CHC [1, 9].

Une approche de « *reverse-engineering* » a été récemment développée afin d’identifier des cibles pertinentes dans des cohortes de patients avec un suivi à long terme, avant de les valider dans des modèles expérimentaux [15]. Pour pousser ce concept et l’adapter à une plateforme de découverte de médicaments et de cibles thérapeutiques, Crouchet *et al*. ont développé un système simple et robuste de culture de cellules humaines hépatiques qui modélise la PLS clinique (décrite ci-dessus) de manière inductible et réversible (système cPLS pour PLS en culture cellulaire). Ce système récapitule les circuits cellulaires impliqués dans la progression de la maladie hépatique et le développement du CHC de manière simplifiée pour toutes les étiologies principales [19–21]. Contrairement aux autres modèles, ce système est basé sur l’utilisation d’une signature cliniquement pertinente et une approche multi-cibles (186 gènes). Il offre la possibilité de valider des cibles thérapeutiques et de découvrir des composés anti-fibrotiques pour la chimioprévention du CHC en testant leurcapacité à réverter le statut de la PLS de mauvais pronostic à un bon pronostic. Ce modèle a récemment été utilisé comme une plateforme innovante et simple de découverte de médicaments en criblant des composés candidats, bio-informatiquement sélectionnés, et déjà approuvés pour une utilisation clinique à long terme sans effets indésirables graves. Le modèle cPLS, combinés à des modèles animaux, a permis la découverte de la nizatidine, un bloqueur du récepteur H2 de l’histamine (HRH2), pour le traitement de la fibrose du foie et la chimioprévention du CHC [21]. Des études mécanistiques ont démontré que la nizatidine prévient le CHC en diminuant l’inflammation du foie, en empêchant le développement de la fibrose et par ses propriétés anticancéreuses directes. La découverte de la nizatidine comme nouveau composé anti-fibrotique pour la prévention du CHC démontre la validité et la relevance du modèle cPLS [21].

Comme décrit ci-dessus, les maladies chroniques du foie induisent chez les patients une reprogrammation épigénétique associée à l’hépatocarcinogenèse. Les modifications épigénétiques, consistant principalement en une hyperméthylation ou une acétylation des histones, entraînent une perturbation de la transcription des gènes. Il a été démontré que les changements épigénétiques sont corrélés à l’induction de la PLS de mauvais pronostic associée au risque de CHC chez les patients [19]. Les protéines à bromodomaines de la famille BET (BET pour *bromodomain and extraterminal domain*) sont des lecteurs de la chromatine qui se lient à l’acétylation des histones et favorisent la régulation des oncogènes en recrutant les complexes de transcription [22] (Figure 1). Les protéines BET sont surexprimées dans plusieurs tumeurs solides et jouent un rôle clé dans l’hépatocarcinogenèse [22]. Il est intéressant de noter que le composé JQ1, un inhibiteur de protéines BET, réverte à la fois le mauvais pronostic de la PLS et de la PES dans le modèle cPLS [19]. En outre, JQ1 a un impact marqué sur la fibrose hépatique dans un modèle de rongeur, restaure le programme transcriptionnel hépatique et empêche le développement du CHC [19]. Les inhibiteurs de BET ont donc été suggérés comme une nouvelle stratégie pour la chimiopréventiondu CHC [23] (Figure 1).

La validation de nouveaux composés anti-fibrotiques pour la chimioprévention du cancer a également nécessité l’établissement de modèles dérivés de tissus de patients, robustes et faciles à utiliser, pour valider les approches thérapeutiques prédisant leur effet en clinique. Pour relever ce défi, le laboratoire de Thomas Baumert a mis au point un système de culture de sphéroïdes générés à partir de tissus de patients, qui comprennent les principaux types cellulaires du foie et reflètent le micro-environnement hépatique. La PLS clinique peut être modélisée dans les sphéroïdes et inversée par le traitement avec des molécules candidates [21]. A l’avenir, nous pouvons imaginer la mise en œuvre de tests cliniques pour des molécules candidates, moins coûteux et plus ciblés, en stratifiant les patients grâce à la combinaison de la plateforme cPLS pour la découverte de cibles thérapeutiques et de nouveaux composés avec des approches de « *reverse-engineering* » et les biomarqueurs du CHC.

### 3 Stratégies de chimioprévention du CHC

Les stratégies de prévention du cancer reposent sur différentes interventions : la prévention primaire, secondaire et tertiaire (Figure 2). La prévention primaire consiste principalement à limiter les facteurs de risque. Il est estimé que 40 à 45 % des cancers sont attribuables à des facteurs de risque évitables, notamment le tabac, l’alcool, la sédentarité et une alimentation déséquilibrée. Alors que l’incidence du cancer augmente dans le monde, la prévention primaire apparaît comme une stratégie clé pour réduire ce fardeau [24, 25]. La prévention primaire du CHC est principalement axée sur la modification et l’amélioration du mode de vie et la vaccination contre le VHB. La prévention secondaire consiste à identifier et à traiter les processus précancéreux ou cancéreux par le biais du dépistage, de la détection précoce et d’un traitement efficace chez les patients déjà exposés aux agents étiologiques. La prévention tertiaire vise à réduire la récidive du cancer après traitement ou la carcinogenèse *de novo* dans un milieu pro-carcinogène [5, 24] (Figure 2).

#### 3.1 Prévention du CHC dans les maladies métaboliques du foie

Environ 25 % de la population mondiale souffre de NAFLD. La NAFLD est fortement associée à un syndrome métabolique incluant l’obésité, la dyslipidémie et le diabète de type 2 [2]. L’obésité et un indice de masse corporelle (IMC) élevé sont directement associés au risque de CHC en favorisant la stéatose, l’inflammation chronique, la résistance à l’insuline et le stress oxydatif [2]. Les directives de l’AASLD recommandent une surveillance du CHC tous les 6 mois chez les patients atteints de NAFLD. Cependant, l’échographie abdominale chez les patients obèses est difficile [6]. Des preuves de plus en plus nombreuses ont montré que cibler les anomalies métaboliques par une intervention nutritionnelle ou pharmaceutique peut être une stratégie efficace pour prévenir le CHC chez les patients obèses et les patients atteints de NAFLD. La principale stratégie préventive pour les patients atteints de NAFLD consiste à modifier leur mode de vie par le biais d’un régime alimentaire approprié et par l’exercice physique, car ils sont bénéfiques pour l’ensemble du spectre pathogène de la NAFLD et réduisent la progression des lésions hépatiques. Les interventions pharmaceutiques sont principalement axées sur la modulation des voies métaboliques et de l’inflammation à l’aide de la metformine et des statines [2, 26]. La metformine est un inhibiteur de la protéine kinase activée par l’AMP (AMPK) utilisé chez les patients diabétiques. Elle a été associée à une réduction du risque de CHC, très probablement en réduisant le stress oxydatif et l’hyperinsulinémie [27, 28]. Les statines sont largement utilisées comme composés hypocholestérolémiants. Cependant, plusieurs études ont suggéré un effet anti-CHC indépendant de cet effet. Il a été démontré que les statines inhibent l’hépatocarcinogenèse en ciblant différents facteurs impliqués dans le développement du CHC, tels que Myc, NF-*κ*B, Akt, en diminuant l’inflammation et en réduisant la fibrogenèse [5]. Les statines peuvent donc avoir un effet protecteur du CHC chez les patients à risque, quelle que soit l’étiologie, mais elles peuvent également être utilisées comme intervention tertiaire pour prévenir les récidives.

#### 3.2 Hépatite B chronique et persistance de l’ADNccc du VHB

L’infection chronique par le VHB est responsable du développement de plus de 50 % des CHC dans le monde. Le programme de vaccination contre le VHB a réussi à réduire le nombre de porteurs chroniques du VHB et est efficace comme prévention primaire du CHC. Cependant, l’infection chronique par le VHB reste une cause majeure de maladie du foie chez les patients non vaccinés, en particulier en Asie du Sud-Est et en Afrique subsaharienne [29]. Selon l’Organisation mondiale de la santé (OMS), plus de 240 millions de personnes sont infectées de façon chronique par le VHB. Ces personnes sont à risque de développer un CHC et représentent une population cible pour la prévention secondaire. Les thérapies antivirales ciblant la réplication du VHB ont été évaluées pour la prévention du CHC. Des études rétrospectives ont rapporté une réduction du risque de CHC après la suppression de la réplication du VHB avec la nouvelle génération d’analogues de nucléotide(s) (l’entécavir et le ténofovir) [30, 31]. Cependant, malgré une réduction significative, le risque de CHC ne peut être éliminé. La surveillance chez les patients atteints d’une infection chronique par le VHB est donc obligatoire. L’intégration de l’ADN du VHB dans le génome de l’hôte ainsi que l’ADN circulaire fermé de manière covalente (ADNccc) sont des caractéristiques uniques du cycle de vie du VHB qui contribuent probablement à la carcinogenèse. Les approches thérapeutiques de nouvelle génération visant à éradiquer complètement le VHB, y compris l’ADNccc viral, pourraient contribuer à améliorer la prévention du développement du CHC à l’avenir [32].

#### 3.3 Hépatite C chronique et risque de CHC persistant après élimination du virus par les DAA

L’infection chronique par le VHC entraîne une fibrose, une cirrhose et des complications associées, notamment une insuffisance hépatique et un CHC. Elle correspond à la principale étiologie du CHC dansles pays occidentaux et au Japon [1]. Dans le monde, 71 millions de personnes sont infectées. Les « baby boomers » représentent la population spécifique présentant les taux les plus élevés d’infection chronique par le VHC et de mortalité liée au CHC, car la plupart des individus ont été infectés avant la découverte du VHC et le développement des techniques de détection modernes [1]. Malgré le développement des DAA qui ont permis d’améliorer l’élimination du VHC, l’incidence du CHC lié au VHC devrait augmenter de façon spectaculaire au cours des prochaines décennies. L’utilisation des DAA est encore entravée par un accès limité et un coût élevé et l’hépatite C chronique est souvent non diagnostiquée car la maladie chronique progresse pendant des décennies sans symptômes. De plus, des études menées dans de grandes cohortes cliniques ont montré que malgré la diminution du risque de CHC après une réponse virologique soutenue, les patients présentant une fibrose hépatique avancée restent à haut risque de CHC [3]. Des études récentes publiées par Hamdane *et al*. ont montré que l’infection chronique par le VHC induit des altérations épigénétiques associées au risque de CHC qui persistent même après la guérison [18]. Les altérations épigénétiques sont donc des cibles intéressantes pour prévenir le CHC chez les patients à risque traités pour une infection par le VHC. De plus, dans une étude de preuve de concept, Jühling *et al*. ont montré que l’inhibition du lecteur de chromatine BRD4 à l’aide du composé JQ1 empêche la progression de la fibrose hépatique vers le CHC en restaurant la reprogrammation transcriptionnelle des gènes épigénétiquement altérés chez les patients [19] (Figure 1). Le CHC induit par le VHC est souvent diagnostiqué à un stade tardif en raison de la longue et asymptomatique progression de la maladie hépatique chronique. Comme le développement d’un vaccin prophylactique contre le VHC reste un défi majeur [33], l’identification des patients à haut risque de CHC est susceptible d’améliorer leur prise en charge et la prévention du CHC.

#### 3.4 Approches de chimioprévention du CHC : exemples d’études cliniques

L’inflammation chronique et la fibrose hépatique sont des facteurs carcinogènes bien établis et constituent donc des cibles attrayantes pour la chimioprévention du CHC [1]. Cependant, il existe un énorme besoin médical non satisfait pour la fibrose hépatique et les thérapies antifibrotiques ne sont pas encore disponibles. D’importants efforts sont déployés pour découvrir des traitements et des cibles thérapeutiques pour traiter les maladies fibrotiques du foie et prévenir le développement du cancer. Plusieurs molécules génériques ciblant l’inflammation et la fibrose sont entrées en essais cliniques et sont également évaluées pour la prévention du CHC (pour une revue, voir [9]). Récemment, il a été démontré que l’utilisation de médicaments anti-inflammatoires non stéroïdiens tels que l’aspirine chez les patients était associée à une diminution de l’hépatocarcinogenèse et du risque de CHC [34]. Cet effet anticancéreux pourrait être dû aux propriétés anti-inflammatoires de l’aspirine par l’inhibition de la signalisation NF-*k*B. Différentes études ont également suggéré une inhibition directe de la P4HA2, une enzyme clé de la synthèse du collagène. Cependant, d’autres études ont montré un effet antiplaquettaire de l’aspirine, ce qui limite son application clinique chez les patients atteints de maladie hépatique chronique [34, 35]. D’autres études sont nécessaires pour comprendre le mécanisme d’action exact.

De multiples voies de signalisation ont été identifiées comme des moteurs de l’hépatocarcinogenèse et de la prolifération cellulaire et sont devenues intéressantes pour la prévention générique du CHC. Par exemple, les inhibiteurs de la voie de la phosphoinositide 3-kinase (PI3K)/AKT/mTOR, le sirolimus ou l’évérolimus, sont actuellement étudiés pour la récidive du CHC dans des essais cliniques de phase III [36]. L’erlotinib, un inhibiteur du récepteur du facteur de croissance épidermique (EGFR), entre également en essais cliniques de phase I à la suite d’études précliniques qui ont démontré que l’erlotinib prévient le développement du CHC *in vivo* et inverse le mauvais pronostic de la PLS associée au risque de CHC [9, 37]. Toutes ces stratégies peuvent être d’un intérêt capital pour prévenir les lésions prénéoplasiques mais aussi pour prévenir la récidive du CHC après résection ou transplantation en ciblant le milieu pro-carcinogène, qui persiste dans le foie lésé, même après élimination des agents viraux.

### 4 Conclusions et perspectives

Le CHC est une cause majeure de décès liés au cancer dans le monde, et son incidence augmentera aucours des prochaines décennies. Malgré des progrès considérables, les traitements curatifs ne sont toujours pas satisfaisants. La grande majorité des patients atteints de CHC sont diagnostiqués à un stade avancé car les populations à risque ne sont pas clairement définies. L’amélioration de la surveillance et du dépistage des patients ainsi que le développement de stratégies de chimioprévention du CHC amélioreront, à l’avenir, la prise en charge des patients et leur pronostic. La promotion d’un changement de mode de vie par les professionnels de la santé peut réduire de manière significative l’incidence du CHC. En outre, la combinaison d’une signature génétique prédictive et/ou de biomarqueurs du CHC aidera à stratifier le risque pour le patient et à établir une stratégie thérapeutique efficace et précise pour la prévention du CHC. Le développement d’un pipeline de découverte de cibles thérapeutiques et de modèles dérivés de patients, associés à des approches de rétroingénierie, contribuera à la mise au point de nouvelles stratégies de chimioprévention. La découverte de composés génériques sûrs réduisant l’inflammation et la fibrose du foie pourrait contribuer au développement de stratégies rentables de chimioprévention du CHC. Comme le CHC peut apparaître à un stade précoce, même dans un foie non fibrosé, en particulier dans le contexte d’une infection chronique par le VHB ou d’une NASH, ces approches peuvent être associées à une intervention spécifique à l’étiologie pour prévenir efficacement le développement du CHC. La chimioprévention devrait améliorer considérablement la prise en charge des patients présentant un risque de CHC.

## Figures and Tables

**Figure 1:**
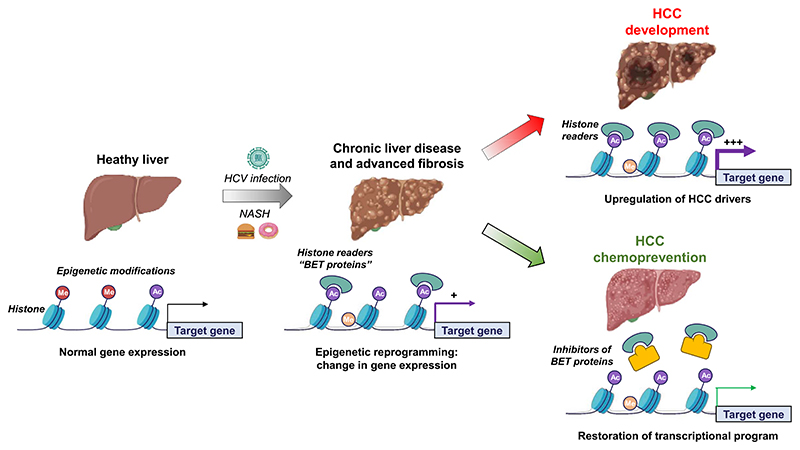
Hepatocellular carcinoma (HCC) chemoprevention by targeting epigenetic modifications induced by chronic viral or metabolic liver disease. Chronic hepatitis C virus (HCV) infection and non-alcoholic steatohepatitis (NASH) induces transcriptional reprogramming through epigenetic modifications (Me = methylation; Ac = acetylation). Bromodomain and extra-terminal motif (BET) proteins are histone readers which bind to acetylated histones and regulate transcriptional initiation and progression. Epigenetic reprograming and BET recruitment drives liver disease progression and HCC development through overexpression of oncogenes or protein regulating cell-cycle. Targeting BET to restore gene expression represents a strategy for HCC chemoprevention as described by Jühling *et al*. [19].

**Figure 2:**
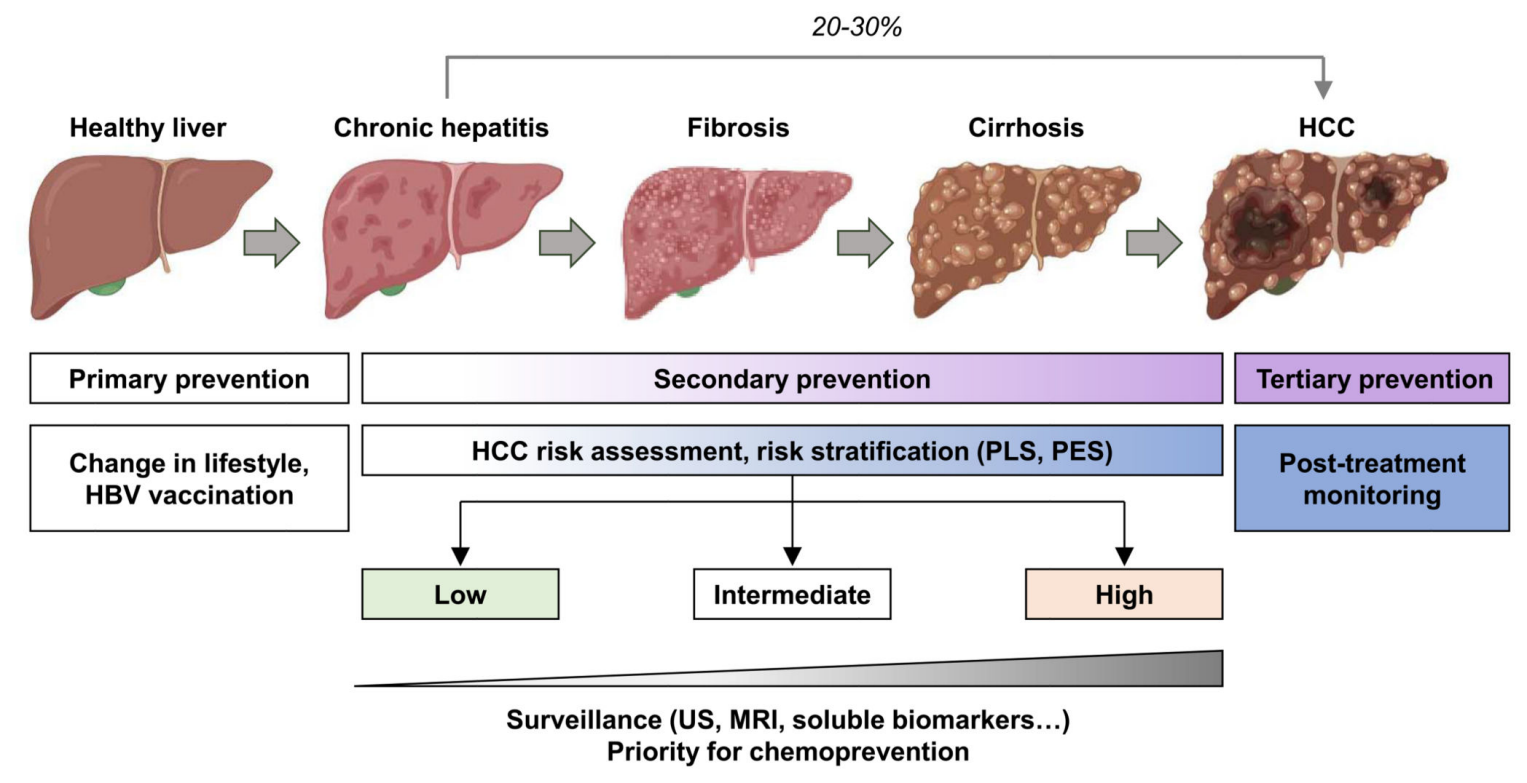
Hepatocellular carcinoma (HCC) screening and prevention. Chronic hepatitis can progress to fibrosis, cirrhosis and lead to HCC. HCC can also arise on non-fibrotic liver. Cancer prevention strategies are based on different interventions: primary, secondary, and tertiary preventions adapted to the clinical context. HCC risk assessment using gene signatures (PLS = prognostic liver signature; PES = prognostic epigenetic signature) may help to prioritize patients for surveillance or chemoprevention (US = ultrasounds; MRI = magnetic resonance imaging).

**Figure 1:**
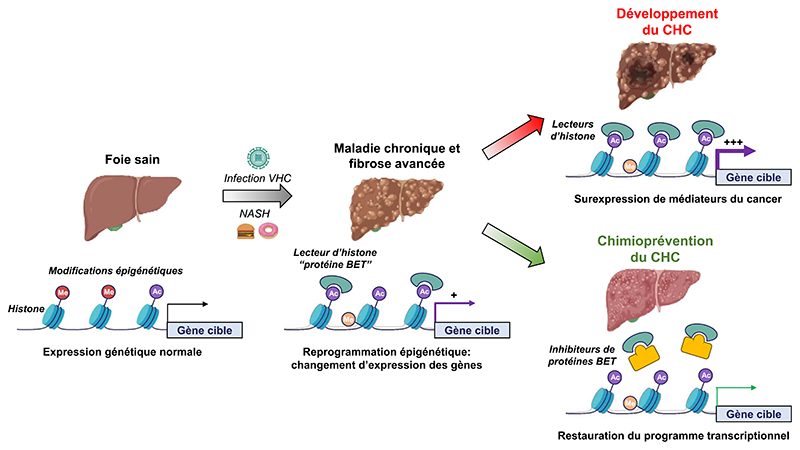
Chimioprévention du carcinome hépatocellulaire (CHC) en ciblant les modifications épigénétiques induites par une maladie hépatique chronique virale ou métabolique. L’infection chronique par le virus de l’hépatite C (VHC) et la stéatohépatite non alcoolique (NASH) induisent une reprogrammation transcriptionnelle par des modifications épigénétiques (Me = méthylation; Ac = acétylation). Les protéines BET (bromodomaine et motif extra-terminal) sont des lecteurs d’histones qui se lient aux histones acétylées et régulent l’initiation et la progression de la transcription. La reprogrammation épigénétique et le recrutement de BET conduisent à la progression des maladies du foie et au développement du CHC par la surexpression d’oncogènes ou de protéines régulant le cycle cellulaire. Le ciblage de BET pour restaurer l’expression des gènes représente une stratégie de chimioprévention du CHC, comme l’ont décrit Jühling *et al*. [19].

**Figure 2:**
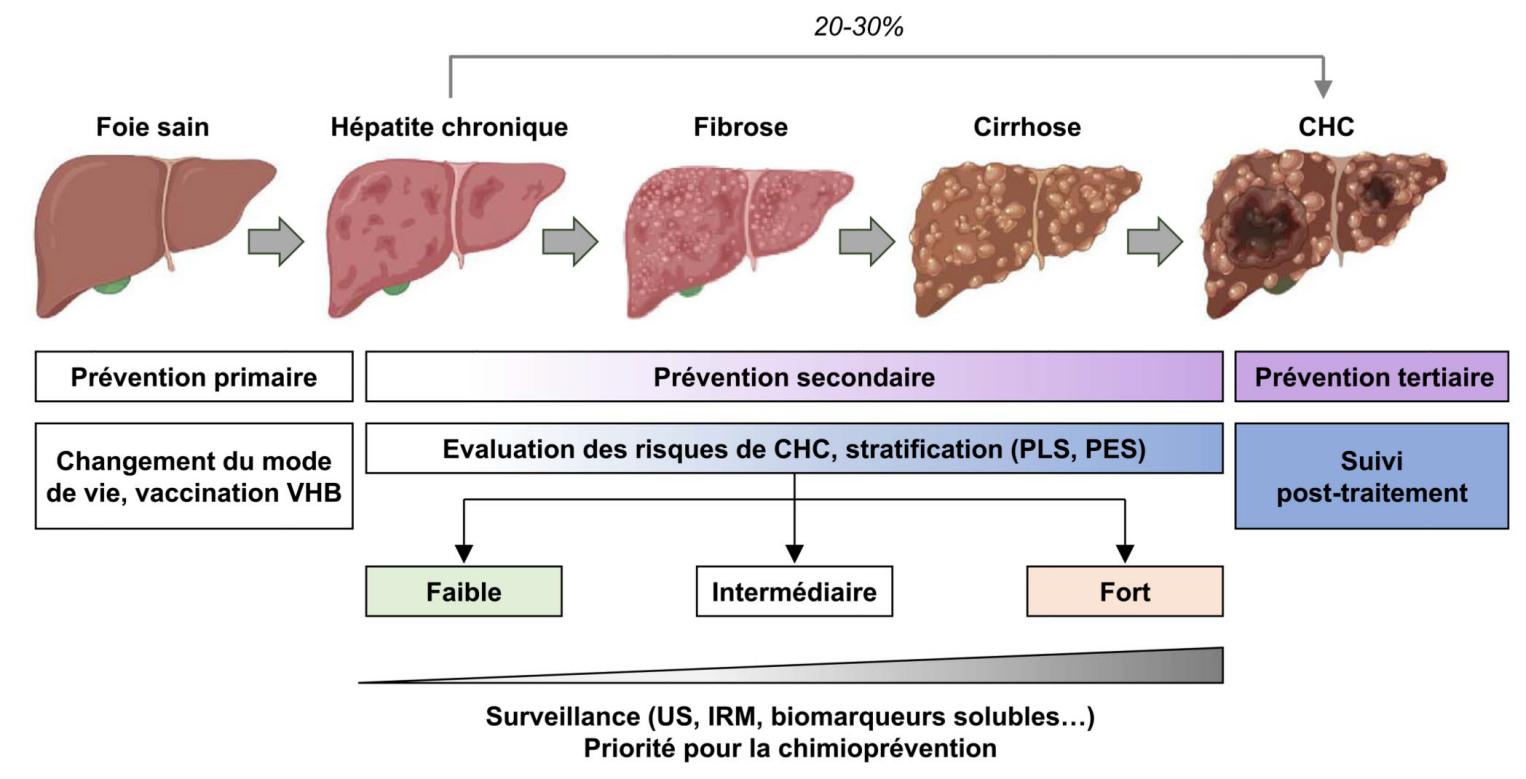
Dépistage et prévention du carcinome hépatocellulaire (CHC). L’hépatite chronique peut évoluer vers la fibrose hépatique, la cirrhose et conduire au CHC. Le CHC peut également se développer sur un foie non fibrotique. Les stratégies de prévention du cancer sont basées sur différentes interventions : préventions primaire, secondaire et tertiaire adaptées au contexte clinique. L’évaluation du risque de CHC à l’aide de signatures génétiques (PLS = signature pronostique du foie; PES = signature pronostique épigénétique) peut aider à stratifier les patients pour la surveillance ou la chimioprévention (US = ultrasons; IRM = imagerie par résonance magnétique).

## Remerciements

Les auteurs remercient le soutien de l’ARC, Paris et de l’Institut Hospitalo-Universitaire, Strasbourg (TheraHCC1.0 et 2.0 IHUARC IHU201301187 et IHUARC2019 ; TFB), l’Union européenne (ERC-AdG-2014-671231-HEPCIR et EU H2020-667273-HEPCAR; TFB), ANRS, Paris (2013/108; TFB), l’US National Institutes of Health (R01CA233794, U19AI12386, R03AI131066; TFB), l’US Department of Defense (W81XWH-16-1-0363; TFB), la Fondation de l’Université de Strasbourg (HEPKIN, Euromedex) et l’Institut Universitaire de France (IUF; TFB). Ce travail a été publié dans le cadre du LABEX ANR-10-LABX-0028_HEPSYS et du Plan Cancer de l’In-serm et bénéficie d’un financement de l’Etat géré par l’Agence Nationale de la Recherche dans le cadre du programme des Investissements d’avenir.
